# Novel Functions of the Neurodegenerative-Related Gene Tau in Cancer

**DOI:** 10.3389/fnagi.2019.00231

**Published:** 2019-08-30

**Authors:** Ricardo Gargini, Berta Segura-Collar, Pilar Sánchez-Gómez

**Affiliations:** ^1^Centro de Biología Molecular, CSIC, Madrid, Spain; ^2^Neurooncology Unit, Instituto de Salud Carlos III-UFIEC, Madrid, Spain

**Keywords:** Tau/MAPT, gliomas, neurodegenerative diseases, cancer, tumor aggressiveness

## Abstract

The analysis of global and comparative genomics between different diseases allows us to understand the key biological processes that explain the etiology of these pathologies. We have used this type of approach to evaluate the expression of several neurodegeneration-related genes on the development of tumors, particularly brain tumors of glial origin (gliomas), which are an aggressive and incurable type of cancer. We have observed that genes involved in Amyotrophic lateral sclerosis (ALS), as well as in Alzheimer’s and Parkinson’s diseases, correlate with better prognosis of gliomas. Within these genes, high *Tau/MAPT* expression shows the strongest correlation with several indicators of prolonged survival on glioma patients. Tau protein regulates microtubule stability and dynamics in neurons, although there have been reports of its expression in glial cells and also in gliomas. However, little is known about the regulation of *Tau/MAPT* transcription in tumors. Moreover, our *in silico* analysis indicates that this gene is also expressed in a variety of tumors, showing a general correlation with survival, although its function in cancer has not yet been addressed. Another remarkable aspect of Tau is its involvement in resistance to taxanes in various tumors types such as breast, ovarian and gastric carcinomas. This is due to the fact that taxanes have the same tubulin-binding site as Tau. In the present work we review the main knowledge about Tau function and expression in tumors, with a special focus on brain cancer. We will also speculate with the therapeutic implications of these findings.

## Introduction

Although the incidence of cancer and neurodegenerative diseases increases with age, there are plenty of evidences of an inverse comorbidity of these two conditions. The inverse association with cancer has been observed for Parkinson’s disease (PD) (Moller et al., 1995; Inzelberg and Jankovic, 2007), as well as for Hungtington’s disease (HD) (Sorensen et al., 1999). Moreover, a decreased risk of tumor incidence has been found after the onset of amyotrophic lateral sclerosis (ALS) (Gibson et al., 2016), although having a cancer diagnosis was not associated with an overall risk of ALS (Fang et al., 2013; Freedman et al., 2014). In the case of Alzheimer’s disease (AD), the inverse comorbidity seems to be maintained in both directions, that is, older people with a history of cancer have a reduced risk of AD, and individuals diagnosed with AD have a decreased risk to develop tumors (Roe et al., 2005, 2010; Driver et al., 2012; Musicco et al., 2013; Ou et al., 2013; Catala-Lopez et al., 2014; Ma et al., 2014). The inverse association between neurodegenerative and oncological diseases appears across many individual types of cancer, even though some studies have reported a positive association of PD with melanoma and prostate cancer (Liu et al., 2011; Kareus et al., 2012). For AD patients there is a more significant decreased risk of lung cancer (Musicco et al., 2013; Ou et al., 2013), and the “protective effect” of previous tumors seems to be greater for smoking related cancers (Driver et al., 2012). Nevertheless, the relationship between smoking and AD is still conflicted. Other question that remains unanswered is what happen with less frequent cancers, like for example brain malignancies, which occur in the same tissue and with the same range of age of the neurodegenerative diseases. The epidemiological evidences in this type of tumors are scarce and sometimes contradictory as they even suggest a direct co-occurrence with AD (Driver et al., 2012). Moreover, a positive correlation has been found between the mortality rate of AD and malignant brain cancer in US (Lehrer, 2010, 2018). Anyhow, these findings support the idea that the two diseases share some environmental and/or genetic risks or that they are governed by similar biological pathways.

Here, we have performed a comprehensive meta-analysis of the genomic and transcriptomic status of genes related to neurodegenerative diseases in brain cancer, particularly in tumors of glial origin (gliomas). Gliomas constitute the most common primary brain tumors and they include astrocytomas, which resemble astrocytes at the histological level, and oligodendrogliomas, with similarities with oligodendrocytes. They are all characterized by their diffuse nature although the WHO (World Health Organization) classify them from the less aggressive grade II, to the grade IV glioblastomas (GBM), whose median overall survival is approximately 15 months (Ceccarelli et al., 2016). Our *in silico* data show a striking inverse correlation between the expression of some of the neurodegenerative genes, especially *Tau/MAPT*, and the clinical progression of gliomas. Here we discuss what could be the implications of these observations and we extend it to the analysis of *Tau/MAPT* in other cancers, where this gene has been associated with resistance to taxanes. The comparative analysis of the biological processes that orchestrate the appearance and the development of gliomas and those that participate in neurodegenerative diseases may potentially lead to a better understanding of both conditions and therefore to the development of more effective therapeutic interventions.

## Materials and Methods

### Analysis of Somatic Mutations and Copy Number Variations

Genetic alterations were analyzed using the TCGA (The Cancer Genome Atlas) data sets. For AD, the genes included were *APP, Tau/MAPT, PSEN1, PSEN2; APOE*, and *GSK3B*; for PD, the genes were *GBA, LRRK2, PARK2 (PRKN), PARK7, PINK1, SNCA*, and *UCHL1;* for *ALS*, the genes were *C9orf72, TARDBP, SOD1, FUS, UBQLN2, and HNRNPA2B1*, which were downloaded from cBioPortal^1^ and UCSC cancer browser TCGA databases^2^.

### Gene Expression Analysis

Information about the progression and the histological types of gliomas was retrieved from the TCGA dataset that includes GBM^3^ and LGG (Lower Grade Gliomas)^4^. These parameters were analyzed together with the expression values of *APP*, *Tau/MAPT, APOE*, *PARK2*, *PARK7*, *PINK1*, *SNCA*, *UCHL1*, *UBQLN2*, and *HNRNPA2B1*^5^^,^^6^. The expression of *Tau*/*MAPT* in different cancers was retrieved from the TCGA PAN-CANCER and GTEx dataset^7^^,^^8^^,^^9^.

### *In silico* Survival Analyses

The expression of *Tau/MAPT* and the follow-up overall survival data from human glioma tumors corresponding to TCGA datasets were downloaded from Xena cancer Browser see text footnote 5 and Gliovis^10^, respectively. Kaplan-Meier survival curves were done following patient stratification using *Tau/MAPT* expression values. TCGA-gliomas LGG + GBM patients (*n* = 690) were stratified in two groups using *Tau/MAPT* median values as thresholds. The significance of the differences in overall survival between groups were calculated using the Log-rank test as Mantel-Cox (Chi square).

Detailed description of how TCGA samples have been processed to generate the dataset of somatic mutations, copy number variations and RNA-seq has been described for LGG dataset [N. Engl. J. Med. 2015 June 25; 372(26):2481–2498] and GBM dataset [Cell. 2013 October 10; 155(2):462–477].

### Primary Glioma Cells

The primary glioma cell lines were obtained by dissociation of surgical specimens from “Hospital 12 de Octubre” (Madrid, Spain), after patient’s written consent and with the approval of the Ethical Committee (CEI 14/023). They belong to the Biobank of that Hospital. Cells were grown in complete media (CM): Neurobasal supplemented with B27 (1:50) and GlutaMAX (1:100) (Thermo Fisher Scientific); penicillin-streptomycin (1:100) (Lonza); 0.4% heparin (Sigma-Aldrich); and 40 ng/ml EGF and 20 ng/ml bFGF2 (Peprotech).

### Mouse Xenograft Assays

Animal care and experimental procedures were performed in accordance to the European Union and National guidelines for the use of animals in research and were reviewed and approved by the Research Ethics and Animal Welfare Committee at our institution (Instituto de Salud Carlos III, Madrid) (PROEX 244/14). For heterotopic xenografts, 1 × 10^6^ cells were resuspended in culture media with Matrigel (1:10, BD) and then subcutaneously injected into athymic nude Foxn1nu mice (Harlan Iberica). For orthotopic xenografts, stereotactically guided intracranial injections in athymic nude *Foxn1*^nu^ mice were performed by administering 0.5 × 10^5^ cells resuspended in 2 μl of Complete Media. The injections were made into the striatum (coordinates: A-P, –0.5 mm; M-L, +2 mm; D-V, –3 mm; related to Bregma) using a Hamilton syringe. Mice were sacrificed at the onset of symptoms.

#### Tumor Tissue Analysis

At the endpoint, subcutaneous tumors or brain tumors were dissected and the tissue was fresh frozen for molecular analysis or fixed o/n in 4% paraformaldehyde (Merck) and embedded in paraffin. Paraffin embedded tissue was cut with a microtome (Leica Microsystems) (3 μm sections) and sections were stained with specific antibodies. For that, slides were heated at 60°C for 1 h followed by deparaffinization and hydration, washed with water, placed into antigen retrieval solution (pressure cooking) in 10 mM sodium citrate pH 6.0 for 10 min. Paraffin sections were permeabilized with 1% Triton X-100 (Sigma-Aldrich) in PBS and blocked for 1 h in PBS with 5% BSA (Sigma), 10% FBS (Sigma), and 0,1% Triton X-100 (Sigma). The following primary antibodies (anti-Tau, Dako A0024; anti-Ki67, Dako M7240 and anti-GFAP, M 0761) were incubated O/N at 4°C. The second day, section was washed with PBS three times prior to incubation with the appropriate fluorescent secondary antibody (anti-mouse and anti rabbit Jackson immunoresearch) for 2 h at room temperature. Prior to coverslip application, nuclei were counterstained with DAPI and imaging was done with Leica SP-5 confocal microscope.

### Western Blot Analysis

For protein expression analysis, a piece of tissue was processed by mechanical disruption in lysis buffer (Tris–HCl pH 7.6, 1 mM EDTA, 1 mM EGTA, 1% SDS, and 1% Triton X100) followed by heating for 15 min at 96°C. Protein content was quantified using BCA Protein Assay Kit (Thermo Fisher Scientific). Approximately 20 μg of proteins were resolved by 10% or 12% SDS-PAGE and they were then transferred to a nitro cellulose membrane (Hybond-ECL, Amersham Biosciences). The membranes were blocked for 1 h at room temperature in TBS-T [10 mM Tris–HCl (pH 7.5), 100 mM NaCl, and 0.1% Tween-20] with 5% skimmed milk, and then incubated overnight at 4°C with the corresponding primary antibody (anti-Tau Calbiochem, 577801 and anti-Actin Sigma, A2228) diluted in TBS-T. After washing 3 times with TBS-T, the membranes were incubated for 2 h at room temperature with their corresponding secondary antibody (HRP-conjugated anti mouse or anti rabbit, Amersham) diluted in TBS-T. Proteins were visible by enhanced chemiluminiscence with ECL (Pierce) using the Amersham Imager 680.

### qRT-PCR Assay

RNA was extracted from the tissue using RNA isolation Kit (Roche). Total RNA (1 μg) was reverse transcribed with PrimeScript RT Reagent Kit (Takara). Quantitative real-time PCR was performed using the Light Cycler 1.5 (Roche) with the SYBR Premix Ex Taq (Takara). The primers used for each reaction were *Tau/MAPT* Fw-GTCGAAGATTGGGTCCCTGG; Rv-GGTCGTAGGGCTGCTGGAA and *HPRT* Fw-TGACACTGG CAAAACAATGCA; Rv- GGTCCTTTTCACCAGCAAGCT. Gene expression was quantified by the delta-delta Ct method.

### Statistical Analysis

Quantitative data, represented as mean ± SD, were compared between groups using two-tailed Student’s t or multiple ANOVA test. One-way ANOVA analysis was used to establish the differences of gene expression data between three groups and was represented as *F* value (F = the ratio of two mean square values). Differences are presented with statistical significance or *p* value (n.s., not significant). For survival study, patients were grouped into two groups with high and low expression of the gene of interest using median values and were analyzed by Kaplan-Meier method and log-rank tests using GraphPad program.

## Results

### Genetic Status of Molecules Associated With Neurogedegenerative Diseases in Gliomas

**FIGURE 1:**
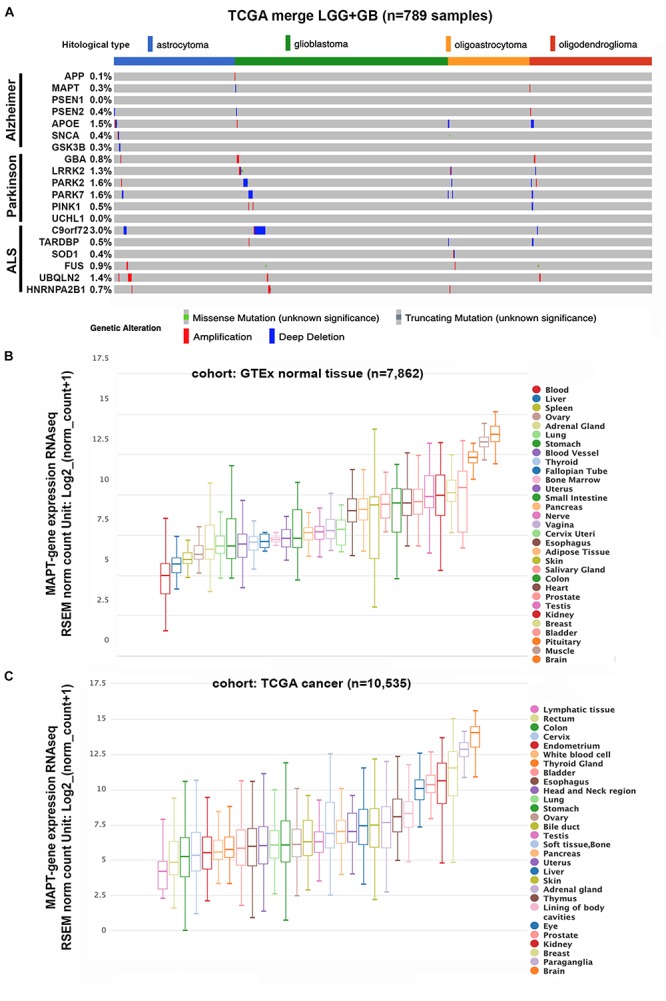
Genetic status and gene expression of neurodegeneration-related genes in cancerous and normal tissues. **(A)** Histogram showing non-silent somatic mutations and copy number variations of genes commonly altered in AD, PD, and ALS on the TCGA cohort of diffuse gliomas (*n* = 789), grouped according to the WHO classification (histological type). **(B,C)** Box plots showing *Tau/MAPT* relative mRNA levels in different normal tissues **(B)** and tumors **(C)**, extracted from TCGA-TARGET-GTEx cohort, with RNAseq-normalized mRNA expression data.

To begin to address if there is a molecular correlation between gliomas and neurodegenerative diseases we analyzed the TCGA merge dataset for LGG and GBM (Ceccarelli et al., 2016) looking for the presence of mutations and copy number alterations in genes associated with AD, PD, and ALS. The genes we chose for AD were *APP* (Amyloid Beta Precursor Protein); *Tau/MAPT*; *PSEN1* (Presenilin 1), *PSEN2* (Presenilin 2), *APOE* (Apolipoprotein E), and *GSK3B* (Glycogen Synthase Kinase 3 Beta). For PD the genes analyzed were *GBA* (Glucosylceramidase Beta), *LRRK2* (Leucine Rich Repeat Kinase 2), *PARK2* (Parkin RBR E3 Ubiquitin Protein Ligase), *PARK7* (Parkinsonism Associated Deglycase), *PINK1* (PTEN Induced Kinase 1), *SNCA* (Synuclein Alpha), and *UCHL1* (Ubiquitin C-Terminal Hydrolase L1). Finally, the genes analyzed for ALS were *C9orf72* (C9orf72-SMCR8 complex subunit); *TARDBP* (TAR DNA Binding Protein), *SOD1* (Superoxide Dismutase 1); *FUS* (FUS RNA Binding Protein), *UBQLN2* (Ubiquilin 2) and *HNRNPA2B1* (Heterogeneous Nuclear Ribonucleoprotein A2/B1). Figure 1A shows that there is a very low frequency of somatic mutations or copy number variations (CNV) in glioma samples for all the genes included in the study. The higher percentage of genetic alterations was found in the *C9orf7* gene (3%), mainly associated with deep deletions that, in any case, are not frequently found in ALS patients. Overall, these results suggest that genetic alterations in genes associated with neurodegeneration are not common drivers of gliomas.

### Expression of Genes Related to Neurodegenerative Diseases in Gliomas

To further assess the involvement of the selected genes in the pathology of gliomas, we analyzed their levels of expression on the same TCGA cohort (Ceccarelli et al., 2016). We summarize in Table 1 the results obtained for those genes that showed significant changes associated with the disease. It is noteworthy that the expression of most of these candidates appear diminished when we compare primary tumors with normal tissue. Moreover, their levels are even lower after tumor recurrence, suggesting that most of these neurodegeneration-related genes are downregulated as the brain pathology progress. By contrast, the opposite trend was observed for the *HNRNPA2B1* gene, which is highly expressed in recurrent tumors compared to primary gliomas or in the latter vs. normal tissue (Table 1).

**TABLE 1:** Correlation of the expression of different neurodegeneration-related genes with the clinical evolution of gliomas.

	**Normal brain/Primary tumor/Recurrent**		**G2/G3/G4 histological grade**		**Survival test statistics**		
	***F*-value anova**	**Expression pattern**	***F*-value anova**	**Expression pattern**	**Media OS (days) high expression**	**Media OS (days) low expression**	**Long-rank value**
**Alzheimer**							
APP	7.92	N>P>R	2.3	G2>G3>G4	2660	1209	6 4
MAPT	9.88	N>P>R	241.1	G2>G3>G4	2975	489	148.8
APOE	6.57	N>P>R	42.2	G2 >G>G4	2660	918	44.2
**Parkinson**							
PARK2	16.27	N>P>R	82.2	G2>G3>G4	2703	880	66.6
PINK1	13.23	N>P>R	77.1	G2>G>3G4	2660	919	45.2
SNCA	15.25	N>P>R	15.1	G2>G>3G4	2235	1137	13.1
UCHL1	10.48	N>P>R	13.9	G2>G>3G4	1233	2000	8.2
**ALS**							
UBQLN2	7.95	N>P>R	104.4	G2>G>3G4	2875	737	106.5
HNRNPA2B1	4.88	N<P<R	4.4	G2>G>3G4	1120	2000	5.1

*Analysis of the expression of genes related to AD, PD, and ALS on the TCGA merged dataset for LGG + GB (Normal brain/Primary tumor/Recurrent tumor, *n* = 701; G2/G3/G4, *n* = 702; Overall survival (OS), *n* = 690 for the merged TCGA dataset (LGG + GB).*

Following the same type of analysis, we evaluated the expression of the selected genes in relation to the histological grade: from LGG (grade 2 and 3) to the aggressive grade 4 GBMs. In agreement with the previous data, the expression of most of the genes decrease as the tumor grade increases. In the particular case of *HNRNPA2B1*, we did not find a direct correlation with the histological grade as the expression levels were higher in grade 3 than in grade 2 or 4 tumors (Table 1).

Finally, we studied what was the implication of the expression of these genes in the survival of glioma patients. Except for *UCHL1* and *HNRNPA2B1*, we found that the high transcriptional levels of the rest of the selected genes are associated significantly with a decrease in tumor burden (Table 1). Taken together, these data supports the notion that the expression of many genes associated with neurodegenerative diseases correlate inversely with the progression of the glioma pathology. The strongest correlation was observed for *Tau/MAPT* so we hypothesized that it must play an important role in gliomas, slowing down or preventing the clinical evolution of these tumors. It is worth mentioning that even though the Tau protein is mostly found in neurons, there are few reports of its expression in glial cells and also in brain tumors of glial origin (Miyazono et al., 1993; LoPresti et al., 1995), reinforcing the need for a deep analysis of the function of this protein in gliomas. Besides, we could get some insights on the oncogenic role of Tau by addressing its significance in other cancers.

### Expression of Tau/MAPT in Cancer

The primary function of Tau protein is the regulation of the dynamic instability of neuronal microtubules during synaptic transmission (Avila et al., 2016; Wang and Mandelkow, 2016). Moreover, tubulin and microtubules are the key components of the mitotic spindle and therefore they control the cell cycle. Actually, microtubule destabilizing and stabilizing components, which include taxanes, have been widely used as anticancer agents as they induce cell cycle arrest and apoptosis (Loong and Yeo, 2014). Because Tau compete with taxanes for the same tubulin-binding domain, a higher expression of this protein has been linked to resistance to these type of compounds, mainly in non-neuronal cancers (Kar et al., 2003). These observations are related to the fact that Tau expression could be expressed in other tissues outside the brain, as it has been already described (Caillet-Boudin et al., 2015). To corroborate these affirmations, we performed an *in silico* analysis of the levels of *Tau/MAPT* mRNA in normal and cancerous tissues in different organs. As expected, the highest expression of this gene occurs in the normal brain. However, elevated levels were also observed in other tissues like muscle, breast, kidney, and prostate (Figure 1B). Remarkably, tumors in those tissues lie among the group of cancers with higher *Tau/MAPT* expression (Figure 1C), suggesting that this gene could have other functions beyond chemoresistance and could be associated with intrinsic properties of the cells in the different organs.

**FIGURE 2:**
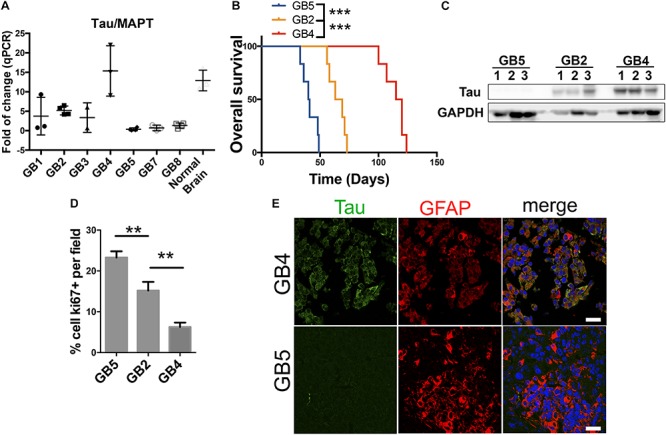
Tau/MAPT expression correlates with less aggressive glioblastomas of PDXs. **(A)** Quantification of Tau/MAPT mRNA expression levels by qPCR in 7 different glioblastomas derived from patients implanted in flanks of nude mice. Normal human brain tissue was used as control. **(B)** Kaplan meier curve of 3 PDXs with different Tau expression levels implanted in the brain of nude mice (*n* = 6 for each group). **(C)** Tau expression levels analysis by western blot of tissue extracts of the experiment described in B using GAPDH as loading control. **(D)** Quantification of Ki67 cells percentage by immunohistochemistry (IHC) of PDXs tissue of samples described in **(B)**. **(E)** Tau and GFAP expression analysis by IHC in tumor tissue of samples described in **(B)**, showing Tau expression in green and GFAP in red, of the PDXs with high and low expression of Tau. ^∗∗∗^*p* value < 0.0001, ^∗∗^*p* value < 0.01. Scale bar 25 μm.

To gain further insight into the relevance of Tau in cancer we analyzed the TCGA cohorts for different cancers and we used the median of *Tau/MAPT* expression to classify tumors into High or Low-Tau. We then studied if there were differences in the overall survival of these two groups. In accordance with our previous data (Table 1), gliomas with high levels of *Tau/MAPT* have a much better prognosis (Table 2). However, the expression of this gene also correlates with an increase in the overall survival of patients with breast cancer, kidney clear cell carcinoma, lung adenocarcinoma and pheochromocytoma/paraganglioma (Table 2). By contrast, we found an inverse correlation between the expression of *Tau/MAPT* and the survival of colon and head and neck cancer (Table 2). However, those tumor have one of the lowest transcriptional levels of this gene (Figure 2). By contrast, it seems that *Tau/MAPT mRNA* is enriched in those cancers in which it has a significant prognostic value, particularly for breast and kidney tumors (Table 2). Collectively, these results suggest that Tau could have an important role in gliomas, but also in other cancers, beyond its competition with microtubule stabilizing agents.

**TABLE 2:** Prognostic value of *Tau/MAPT* in different cancers.

	***N***	**Tau(MAPT) exp (Median)**	**Pro-survival**	**Median OS (days) high-Tau**	**Median OS (days) low-Tau**	**Survival *p* value**	**Survival log-rank value**
Acute myeloid leukemia	132	4.52	n.s				
Adrenocortical cancer	79	6.91	n.s				
Bile duct cancer	**55**	5.65	n.s				
Bladder cancer	429	4.91	n.s				
Breast cancer	**1200**	**10.72**	**Yes**	**3736**	**3472**	**<0.0001**	**21.32**
Cervical cancer	296	4.46	n.s				
lightred							
Colon cancer	**315**	**6.83**	**No**	**1881**	**3041**	**0.0089**	**6.83**
Endometrioid cancer	188	4.61	n.s				
Esophageal cancer	196	4.74	n.s				
Glioma	**690**	**13.27**	**Yes**	**2875**	**489**	**<0.0001**	**148.8**
Head and neck cancer	**562**	**5.17**	**No**	**1090**	**1732**	**0.0073**	**7.19**
Kidney clear cell carcinoma	**604**	**10.47**	**Yes**	**Undefined**	**2090**	**<0.0001**	**29.67**
Large B-lymphoma	47	3.25	n.s				
Liver cancer	417	6.63	n.s				
Lung adenocarcinoma	**564**	**5.31**	**Yes**	**1632**	**1423**	**0.0334**	**4.52**
Lung cancer	1109	5.14	n.s				
Melanoma	462	6.57	n.s				
Mesothelioma	85	7.48	n.s				
Ovarian cancer	307	5.16	n.s				
Pancreatic cancer	182	6.15	n.s				
Pheochromocytoma/Paraganglioma	**185**	**12.13**	**Yes**	**Undefined**	**Undefined**	**0.0383**	**4.29**
Prostate cancer	549	9.55	n.s				
Rectal cancer	103	3.99	n.s				
Sarcoma	265	6.15	n.s				
Stomach cancer	421	4.92	n.s				
Testicular cancer	139	5.21	n.s				
Thymoma	121	7.23	n.s				
Thyroid cancer	571	4.91	n.s				
Uterine carcinosarcoma	56	6.24	n.s				

*Analysis of *Tau/MAPT* gene expression on the TCGA dataset for the different types of tumors. The median of *Tau/MAPT* expression was used to separate the high- and low-Tau groups and to calculate the overall survival (OS). Log-rank (Mantel-Cox) analysis was performed to determine the Pro-Survival value of *Tau/MAPT* expression. Bold words show relevant values for *Tau/MAPT*. *N*, number of patients enrolled. n.s., non-significant.*

### Tau/MAPT Is Expressed on Glioma Cells and Correlates With a Less Aggressive Behavior of These Tumors

The data presented here suggest that *Tau/MAPT* is expressed in certain types of cancers, especially in the brain. Moreover, it allow us to hypothesize that, in some cases, the expression of this gene could be a marker of the less undifferentiated tumors (those that show a better clinical behavior) that get lost during the progression of the disease. In order to gain insight into the possible role of Tau in cancer, particularly in gliomas, we analyzed its expression in a panel of patient-derived-xenografts (PDXs). In these subcutaneous xenografts, in the absence of *Tau/MAPT* positive neurons, we observed that there is a variability in the transcriptional levels of this gene (Figure 2A). We then chose 3 of these PDX, with high, intermediate and low expression of *Tau/MAPT*, and we injected the dissociated cells into the brain of immunodeficient mice. We observed a striking inverse correlation between the amount of Tau and the tumor growth (Figures 2B,C). In fact, the more aggressive tumors show higher proliferative index (Ki67 staining) (Figure 2D). Interestingly, the analysis of the dissected GBM xenografts confirmed that Tau is expressed in a high percentage of tumor cells (co-stained with a GFAP antibody) in the Tau-high PDX, whereas there is very little expression in the Tau-low PDX (Figure 2E). Altogether, these results confirm the specific expression of Tau in glioma cells. Moreover, they suggest that the levels of this protein correlate inversely with the progression of this pathology.

## Discussion

Epidemiological evidences point toward an inverse comorbidity of neurodegenerative diseases and cancer. This phenomenon could be influenced by environmental factors and drug treatments. However, genetic and molecular pathways should have an important contribution as well. In fact, the two maladies are characterized by opposing cellular behavior in relation to cell growth and survival. In the case of AD, for example, a pathogenesis model has been proposed based on the up-regulation of tumor suppressors (Blalock et al., 2004). In that sense, p53, which promotes apoptosis and protects against cancer, is overexpressed in the central nervous system (CNS) of patients diagnosed with a neurodegenerative disease (Behrens et al., 2009). Others have found that several genes and pathways are upregulated in CNS disorders but downregulated in cancer and the other way round (Ibanez et al., 2014). This particular study has confirmed the dual role for the p53 pathway as well as for two other candidates, the enzyme Pin1 and the Wnt pathway, which are overexpressed or activated in most cancers but downregulated in AD (Behrens et al., 2009). More recently, another functional analysis has suggested that proteosomal, protein folding and mitochondrial processes are regulated oppositely in AD and lung cancer (Sanchez-Valle et al., 2017). The same study found a significant number of immune system and oxidative-phosphorylation-related genes that were deregulated in the same direction in AD and aggressive gliomas. More recently, it has been proposed that GBM-secreted CD44 could induce neuronal degeneration through the activation of Tau pathologies in the brain (Lim et al., 2018). These findings suggest a positive correlation between the last two diseases, something that had already been proposed (Lehrer, 2010, 2018). However, there are no robust epidemiological data on the incidence of gliomas in AD patients or vice versa, probably due to the low frequency of these tumors. Moreover, there are also discrepancies at the molecular level as other authors have found that the ERK/MAPK (associated with cell proliferation and survival) and the Angiopoietin (associated to angiogenesis) signaling pathways are oppositely regulated in the two diseases (Liu et al., 2013). Given this controversy we decided to perform a comprehensive characterization in gliomas of the genetic and transcriptomic status of genes associated with neurodegenerative diseases.

Despite the lack of genetic alterations of our panel of neurodegenerative genes in gliomas, we have observed an important correlation of their transcriptional expression with the clinical behavior of this type of cancer. It is true that almost all the genes tested have a higher expression in normal tissue compared to primary gliomas, nevertheless this could reflect that they are enriched in neurons in comparison to glial cells. However, the most relevant data is that their transcriptional levels diminish in parallel with the clinical evolution of the disease: after recurrence and during the progression of gliomas from low to higher grade tumors. Among the studied genes, we decided to focus on *Tau/MAPT*, as it shows the strongest correlation with the clinical evolution of gliomas. We performed an *in vivo* analysis, with ortho and hetherotopic glioma xenografts, which confirmed that Tau protein is truly expressed in the astrocytic tumor cells. Moreover, we validated the inverse correlation between the Tau and glioma growth. Therefore, our findings suggest that *Tau/MAPT*, and some other genes whose alterations are linked to neurodegenerative diseases, are enriched in the less aggressive gliomas and they probably need to be downregulated for these tumors to progress. This phenomenon could explain their positive correlation with survival in almost all the cases analyzed.

It is worth mentioning that there have been previous reports of deregulations (mutations and copy number losses) observed in *PARK2* in GBM (Veeriah et al., 2010), which argue in favor of a possible tumor suppressor function of this gene in such tumors. Still, our meta-analysis indicates a lower frequency of *PARK2* deletions in the joint cohort of gliomas. However, we only accounted for those CNV that appear in homozygosity, which could explain why we found a reduced percentage of gliomas with these deletions. Nevertheless, our transcriptomic data further supports that this protein could be inhibiting glioma growth as it has been suggested by others (Yeo et al., 2012; Viotti et al., 2014; Gupta et al., 2017). Regarding Tau, it has been recently reported that families affected by genetic tauopathies have a higher risk of developing cancer (Rossi et al., 2018). However, our analysis has found a very small percentage of gliomas (0.3%) with Tau alterations (amplifications and deletions).

Although Tau is highy expressed in neuronal axons, there were evidences of its presence in glial cells (LoPresti et al., 1995) and even in gliomas (Lopes et al., 1992; Miyazono et al., 1993). Moreover, the levels of Tau had been recently associated with LGG survival rates (Zaman et al., 2019), although its role in brain cancer was largely unknown. Our results suggest that gliomas need to lose Tau expression in order to progress into a more aggressive entity. In AD and other tauopathies, it has been postulated that changes in the expression of the different *Tau/MAPT* splicing isoforms might facilitate the neurodegeneration. Besides, Tau becomes increasingly phosphorylated in AD, which is the first step in the formation of toxic aggregates of the protein (gain of toxic function). The phosphorylation and subsequent aggregation of Tau compromises its microtubule-stabilizing functions (loss of physiological function), favoring the evolution of the pathology. In agreement with the loss-of-function model, several groups have reported behavioral changes and neurogenesis in aged Tau knockout mice (Pallas-Bazarra et al., 2016). Therefore, we could postulate that Tau expression and/or function might be lost during the progression of both, gliomas and AD, which might contribute to explain the correlation between the two diseases observed by certain authors. Nevertheless, the mechanism for Tau inhibition could be seemingly different: gene expression regulation in gliomas and posttraductional modifications in AD and other Tauopathies.

In relation to Tau function, our results suggest that the stabilization of the microtubule network might be needed in the earliest stages of gliomas, but it would be lost as the tumors progress. Tau overexpression in immortalized cell lines induces a plethora of changes in the location and trafficking of different organelles, including mitochondria and endosomes. This leads to a decrease in cell proliferation (Ebneth et al., 1998), which could be associated with the reduction in tumor growth that we have observed in the Tau-high gliomas. Moreover, changes in the dynamics of the microtubular cytoskeleton might have important implications in the motility of the tumor cells, affecting their invasive capacity. In adition, the microtubules participate in the process of epithelial-to-mesenchymal transition (EMT), which also occur in gliomas and has been associated with an increase in the aggressivity of these tumors (Bhat et al., 2013). In fact, the higher expression of Tau/MAPT in lower degree gliomas suggest that it could be an accumulation of this protein in the so-called Proneural tumors, which have a better prognosis and tend to suffer a process of mesenchymalization after tumor recurrence (Bhat et al., 2013). Interestingly, we have shown that Proneural gliomas are more prone to invasion whereas mesenchymal tumors are more angiogenic (Garcia-Romero et al., 2016). Therefore, we could speculate that changes in Tau expression could affect at the same time the EMT and the invasion capacity of the glioma cells. In fact, a recent article suggests that Tau downregulation in a glioma cell line leads to a decrease in cell motility (Breuzard et al., 2019). Future experiments will help to determine if Tau is an active or passive actor on these phenotypes.

Appart for the control of the microtubule dynamics, Tau has been implicated in other functions like maintenance of the RNA/DNA integrity and the nuclear structure. In addition, Tau contains several domains with a broad pattern of binding partners, including other cytoskeletal molecules that could participate in the regulation of protein transport (Medina et al., 2016; Wang and Mandelkow, 2016). Besides, Tau interacts with a plethora of kinases, phosphatases, chaperones and membrane proteins (Mandelkow and Mandelkow, 2012) and many of those could be Tau partners in cancer. Indeed, it has been shown that in prostate cancer cells, Tau binds to PI3K (phosphatidylinositol 3-kinase) (Souter and Lee, 2009). Furthermore, it has been shown to activate MAPK (Mitogen-activated protein kinase) in response to NGF (nerve growth factor) and EGF (epidermal growth factor) (Leugers and Lee, 2010). Both, PI3K and MAPK are important regulators of glioma growth and survival (Ceccarelli et al., 2016). More recently, Tau deletion has been linked to defects in the insulin signaling pathway in the brain (Marciniak et al., 2017). This could explain why patients with AD appear to be insulin resistant (Talbot et al., 2012). Moreover, it could be the cause of the repression of the orthotopic growth and vascularization of gliomas in genetic mouse models of AD (Paris et al., 2010). Alternatively, we could hypothesize that the decrease in Tau function could participate in the brain neuroinflammation observed in AD models and that this phenomena could favor glioma growth (Sanchez-Valle et al., 2017; Laurent et al., 2018). Indeed, higher levels of inflammatory markers have been observed in the brains of AD mice, which are more prone to develop tumors in the presence of carcinogens (Serrano et al., 2010). Future experiments will help to solve these apparent discrepancies in the frequency of cancer in AD mouse models.

Another important corollary of our study is the possibility that microtubules-stabilizing compounds could imitate Tau and hinder glioma growth. Among these molecules the most commonly used are the taxanes, including paclitaxel and docetaxel, which serve as chemotherapy agents in different cancers. Although they can reduce glioma growth *in vitro*, the clinical trials were not successful due to limitations in their ability to cross the blood brain barrier (Glantz et al., 1999). However, there have been promising results with the next-generation of taxanes like epothilone D and cabazitaxel, which can penetrate the brain and have good anti-tumor activity in mouse glioma models (Semiond et al., 2013). Cabazitaxel has been evaluated with a limited success in a phase II study for temozolomide refractory gliomas (NCT01740570 and NCT01866449) and now is being investigated clinically in combination with cisplatin.

High tau mRNA expression has been associated with a more favorable prognosis in breast cancer (Shao et al., 2010; Baquero et al., 2011). These results were confirmed in the present study (Table 2). Moreover, we have extended these results to other unreported cancers, like Kidney Clear Cell Carcinoma. Interestingly, it has been proven that Tau expression has a predictive value for estrogen receptor (ER)-positive breast cancer. In those tumors high levels of Tau are considered a surrogate marker for endocrine-sensitive but otherwise chemotherapy-resistant samples (Pentheroudakis et al., 2009; Shao et al., 2010; Bonneau et al., 2015). By contrast, low Tau expression identifies a subset of ER-positive breast cancers that have poor prognosis when treated with tamoxifen alone, although they may benefit from chemotherapy with taxanes (Rouzier et al., 2005; Andre et al., 2007; Pentheroudakis et al., 2009; Tanaka et al., 2009; Shao et al., 2010; Bonneau et al., 2015). Similarly, Tau-negative expression has been related to a favorable response to Paclitaxel treatment in gastric (Mimori et al., 2006; Wang et al., 2013; He et al., 2014; Yu et al., 2014) and bladder cancer (Wosnitzer et al., 2011). Translating these results into gliomas, our findings suggest that Tau-positive expression could identify a subset of tumors with slowest progression (including those with a higher sensitivity to the standard chemo-radiation therapy). By contrast, low-Tau expression could be a marker of a subset of gliomas that has a poorer prognosis. However, those tumors might benefit from chemotherapies with taxane-derivatives, like cabazitaxel. Therefore, it will be interesting to quantify the value of Tau expression as a surrogate marker in the clinical trials with this compound.

Increasing efforts have focused on the physiological vs. the pathological properties of Tau, investigating mechanisms of neuronal dysfunction attributed to loss-of-normal or gain-of-toxic Tau function in AD and other neurodegenerative pathologies. However, despite the evidences that support a novel role for Tau in cancer, we know very little about the mechanisms that control the expression and/or the function of this protein in tumor cells or in their microenvironment. The results presented here underlines the relevance of Tau in cancer, beyond its capacity to compete with chemotherapy compounds. We propose that this protein might serve as a brake in the evolution of the pathology of several tumors, especially in gliomas and certain breast and kidney cancers, where it is highly expressed. We believe that a profound characterization of the expression and function of Tau in normal and tumorigenic glial cells is needed. These studies will probably uncover novel aspects of the biology of this protein that could help to unravel the etiology of several cancers and neurodegenerative diseases.

## Data Availability

The datasets generated for this study are available on request to the corresponding author.

## Author Contributions

RG and PS-G conceived the study. All authors developed the data analysis, wrote and criticized the manuscript, reviewed the literature, and revised and approved the manuscript. RG and BS-C performed the *in vivo* analysis of glioma growth.

## Conflict of Interest Statement

The authors declare that the research was conducted in the absence of any commercial or financial relationships that could be construed as a potential conflict of interest.

## Footnotes

1 http://www.cbioportal.org

2 https://tcga.xenahubs.net

3 https://www.cell.com/cell/pdfExtended/S0092-8674(13)01208-7

4 https://www.nejm.org/doi/full/10.1056/NEJMoa1402121

5 https://xenabrowser.net

6 https://www.ncbi.nlm.nih.gov/pubmed/24120142

7 https://www.ncbi.nlm.nih.gov/pubmed/29022597

8 https://www.ncbi.nlm.nih.gov/pubmed/25954001

9 https://www.ncbi.nlm.nih.gov/pubmed/30881377

10 http://gliovis.bioinfo.cnio.es
